# Early detection and staging of spontaneous embryo resorption by ultrasound biomicroscopy in murine pregnancy

**DOI:** 10.1186/1477-7827-12-38

**Published:** 2014-05-10

**Authors:** Luis E Flores, Thomas B Hildebrandt, Anja A Kühl, Barbara Drews

**Affiliations:** 1 Department Reproduction Management, Leibniz Institute for Zoo and Wildlife Research (IZW), Alfred-Kowalke-Str. 17, 10315 Berlin, Germany; 2 Charité – Department of Medicine I for Gastroenterology, Infectious Disease and Rheumatology, Research Center ImmunoSciences / Universitätsmedizin Berlin, Campus Benjamin Franklin, Hindenburgdamm 30, 12203 Berlin, Germany

**Keywords:** Embryonic failures, Yolk sac, Reichert’s membrane, Embryo resorption, Ultrasound biomicroscopy, Murine pregnancy, Placenta

## Abstract

**Background:**

Embryo resorption is a major problem in human medicine, agricultural animal production and in conservation breeding programs. Underlying mechanisms have been investigated in the well characterised mouse model. However, post mortem studies are limited by the rapid disintegration of embryonic structures. A method to reliably identify embryo resorption in alive animals has not been established yet. In our study we aim to detect embryos undergoing resorption in vivo at the earliest possible stage by ultra-high frequency ultrasound.

**Methods:**

In a longitudinal study, we monitored 30 pregnancies of wild type C57BI/6 mice using ultra-high frequency ultrasound (30-70 MHz), so called ultrasound biomicroscopy (UBM). We compared the sonoembryology of mouse conceptuses under spontaneous resorption and neighbouring healthy conceptuses and correlated the live ultrasound data with the respective histology.

**Results:**

The process of embryo resorption comprised of four stages: first, the conceptus exhibited growth retardation, second, bradycardia and pericardial edema were observed, third, further development ceased and the embryo died, and finally embryo remnants were resorbed by maternal immune cells. In early gestation (day 7 and 8), growth retardation was characterized by a small embryonic cavity. The embryo and its membranes were ill defined or did not develop at all. The echodensity of the embryonic fluid increased and within one to two days, the embryo and its cavity disappeared and was transformed into echodense tissue surrounded by fluid filled caverns. In corresponding histologic preparations, fibrinoid material interspersed with maternal granulocytes and lacunae filled with maternal blood were observed. In later stages (day 9–11) resorption prone embryos were one day behind in their development compared to their normal siblings. The space between Reichert’s membrane and inner yolk sac membrane was enlarged The growth retarded embryos exhibited bradycardia and ultimately cessation of heart beat. Corresponding histology showed apoptotic cells in the embryo while the placenta was still intact. In the subsequent resorption process first the embryo and then its membranes disappeared.

**Conclusions:**

Our results provide a temporal time course of embryo resorption. With this method, animals exhibiting embryo resorption can be targeted, enabling the investigation of underlying mechanisms before the onset of total embryo disintegration.

## Background

Embryo resorption is not only a major problem in human reproductive medicine [1,2] but also in agricultural animal production [3] and in conservation breeding programs (reviewed in Andrabi and Maxwell [4]). The underlying mechanisms are manifold and include chromosomal anomalies [5], placental insufficiency [6] and disturbances in the feto-maternal immune tolerance [7].

Studies on embryo resorption in humans are restricted due to ethical reasons. Here, the mouse serves as a biomedical model. Its small size and its fast reproduction mode enable large-scale studies. Moreover, knock-out strains allow for a straight forward functional analysis of genes involved in the establishment of pregnancy [8,9]. Up to date, studies on embryo resorption are performed mainly post mortem [10-12] and pregnant animals are sacrificed at certain days of pregnancy to determine the resorption rate. The resorption rate is defined as the ratio between the number of resorptions and the number of normal implantations [13]. The inaccuracy of this method is rooted in the uncertain time point of embryonic death. Counting the number of resorptions on e.g. day 12 post ovulation might very likely include embryos that died considerably earlier in pregnancy. To yield a reliable result on the actual rate of resorption in post mortem studies, high animal numbers are needed to systematically evaluate every day of pregnancy. If animals are sacrificed randomly, the identification of the cause of embryo death and subsequent resorption is difficult due to the rapid disaggregation of embryonic structures. In studies on early embryo loss, among hundreds of implantation sites, only one conceptus was either not completely normal, or completely destroyed but in a state of early resorption [10]. This fact is a major concern for the investigation of feto-maternal immune interactions, where cause and effect are especially difficult to distinguish.

Ultra-high frequency ultrasound or so called “Ultrasound Biomicroscopy, (UBM)” can depict structures smaller than 0.1 mm and enables *in vivo* monitoring of prenatal development in small animals. UBM has been used to high-lighten the peculiarities of the long pregnancy of the naked mole rat [14] and to describe embryo development, embryo resorption and corpus luteum regression in the European brown hare [15,16]. In mouse development, UBM has been employed to establish growth graphs for determination of gestational age [17] and to describe the gross development of the mouse embryo [18-20]. Phoon et al. have analyzed embryo cardiovascular function by UBM [21-23]. Most recently, UBM has been used to evaluate the effect of defined quantitative trait loci on embryo lethality in a mouse model of interspecific recombinant congenic strains [20,24]. In these studies, the number of dead and living embryos was assessed on defined days of gestation. To date, there is no *in vivo* description of the process of murine embryo resorption.

In our study, we aimed to identify conceptuses undergoing spontaneous resorption at the earliest possible stage by UBM. The dynamics between mother, placenta and embryo of implantation sites under resorption were compared to their normally developing littermates. Different stages of embryo resorption were exemplarily evaluated by histology to verify UBM data. A time line of the process of spontaneous murine embryo resorption was established. On the basis of this study, growth retarded embryos and embryos that undergo resorption can be identified *in vivo*.

## Methods

### Animals

All experimental work on live animals complied with our institutional and governmental regulations (Tierschutz-Versuchstierordnung). The institutes committee for animal welfare and ethics and the State Office of Health and Social Affairs Berlin approved the experimental design (letter 03.11.2010) in accordance with §8a of the German law of animal welfare (Tierschutzgesetz). Mice from the inbred C57BL/6 strain were obtained from Harlan Laboratories, Rossdorf, Germany. A total of 30 females and 4 males were kept in open top-wire cages under a 12 h light–dark regime with food and water *ad libitum*. A microchip implant was used for individual identification (Hong Teng Technology, Guangzhou, China). Mice were mated for a period of 3 days in breeding groups comprised of 4 females and one male.

### Examination of pregnancies

Successful mating was confirmed by the presence of a vaginal plug after establishment of breeding groups. In some animals, a vaginal plug could not be detected but pregnancy was confirmed by ultrasound. With the exception of four animals (ID 4, 6, 7 and 13) ultrasound examinations were performed on a daily basis starting on day 4 after establishment of the breeding groups. Pregnancy could be confirmed earliest by the ultrasonographic visualization of decidualized implantation sites on day 5 after establishment of the breeding groups. If implantation sites were detected one or two days later (day 6 and 7 after establishment of breeding groups), we assumed that mating had occurred later, too. Consequently, the day of the first visualization of the implantation sites was always referred to as day 5 of pregnancy. If no pregnancy could be confirmed 8 days after establishment of breeding groups, the animal was considered non-pregnant and was mated again. For the ultrasound examination, an Ultrasound Biomicroscope (Vevo 2100, Visual Sonics, Toronto, Canada) equipped with a 30-70 MHz transducer (MS700) was used. The ultrasound settings were standardized as follows: frequency–50 MHz, power 100%, gain– 30 db. Prior examination, mice were anesthetized using inhalation anesthesia via a mask. 5% isoflurane (CP-Pharma, Burgdorf, Germany) was delivered for induction and 1.5%-2% for maintenance with an oxygen flow of 1 l/min. To avoid hypothermia, animals were placed on an electric heating mat and the ultrasound gel for the transducer was warmed in a water bath before use. To ensure optimal image quality, the abdominal hair was removed using commercial chemical hair removal gel (Veet, Köln, Germany). In the course of the ultrasound examination the number of conceptuses in each uterine horn was determined. The viability and the staging of the conceptuses was evaluated according to biometric measurements and morphologic parameters. Biometric measurements included the size of the implantation site, the size of the embryonic cavity (EC), the crown-rump-length (CRL) the biparietal distance (BPD) and placental measurements. The size of the implantation site was determined by averaging two perpendicular maximal diameters. The size of the EC was measured in its maximal diameter. Morphologic parameters were the differentiation of decidua basalis (DB) and decidua capsularis (DC), formation of the ectoplacental cone (EPC), the presence of embryonic membranes and the presence and quality of heartbeat.

### The duration of the scanning procedures ranged from 10 to 20 minutes per individual

Relevant ultrasound data was recorded for each conceptus. The resorption process was documented by UBM and the respective animals were sacrificed at defined days after the onset of embryo resorption for histological analysis.

### Whole conceptus collection and processing

For the collection of normal conceptuses and conceptuses under resorption, the mouse was euthanized by cervical dislocation and the reproductive tract was removed. The number of healthy embryos and resorption sites was counted and photographed. The uterus was examined by UBM in a water bath with a 0.9% physiological saline solution (Braun, Melsungen, Germany) to verify the *in vivo* ultrasound data. A solution of 4% paraformaldehyde in 1x phosphate buffered saline with a pH 7.4 was used for fixation. A standard protocol for paraffin embedding and sectioning was followed. Sections had a set 3–5 μm thickness. For morphological analysis sections were stained with hematoxylin and eosin (H&E) following standard protocol.

## Results

In total, we followed 30 pregnancies in 30 different females by longitudinal UBM examinations. The mean number of implantation sites per animal was 7.5 with a range of 1 to 12 implantations per animal. Embryo resorptions were identified between day 7 and 13. In total, 23 resorptions (R1-R23) were detected in 15 pregnancies. Taking all 30 pregnancies into account this resulted in a resorption rate of 10.22% (N = 225 normal implantations versus 23 resorptions). To verify and supplement the ultrasound data, an exemplarily collection of resorption sites for histological analysis was obtained. The time points of detection and collection of resorption are summarized in Table 1. In three animals, older resorption sites (R9, R10, R12 and R14) that had occurred earlier in pregnancy were collected together with a fresh resorption site that was identified later in pregnancy (R11, R13 and R15).

**Table 1:** Ultrasonographic detection of embryo resorption and day of collection

**Mouse ID**	**ID of 1**^**st **^**resorption**	**Detection of 1**^**st **^**resorption (day)**	**ID of 2**^**nd **^**resorption**	**Detection of 2**^**nd**^**resorption (day)**	**ID of 3**^**rd **^**resorption**	**Detection of 3**^**rd**^**resorption (day)**	**Number of resorptions per animal**	**Collection of resorption sites (day)**
1	R1	d7	R2	d7			2	8
2	R3	d7					1	8
3	R4	d7					1	8
4	R5	d8	R6	d8			2	9
5	R7	d8					1	9
6	R8	d8					1	9
7	R9	d7	R10	d7	R11	d9	3	9
8	R12	d7	R13	d9			2	9
9	R14	d8	R15	d9			2	10
10	R16	d9	R17	d9			2	11
11	R18	d10					1	10
12	R19	d9	R20	d9			2	10
13	R21	d12					1	12
14	R22	d12					1	12
15	R23	d13					1	13

### The number and location of the conceptuses was determined in every ultrasound examination

On day 5, we underestimated the total number of implantation sites by two in one animal, and by one in four animals. On day 6, the number of conceptuses in these animals was corrected and confirmed in subsequent examinations. On day 7, we counted one embryo twice in one animal. The localization of the conceptuses in the right and left uterine horn respectively was always correctly determined with the exception of one embryo on day 6. This mistake was also corrected one day later during the next examination. Apart from these cases, the number and position of embryos during *in vivo* examinations were consistent with the number and position of the conceptuses and resorption sites as derived from the exteriorized uterus and water bath examinations.

**Figure 1:**
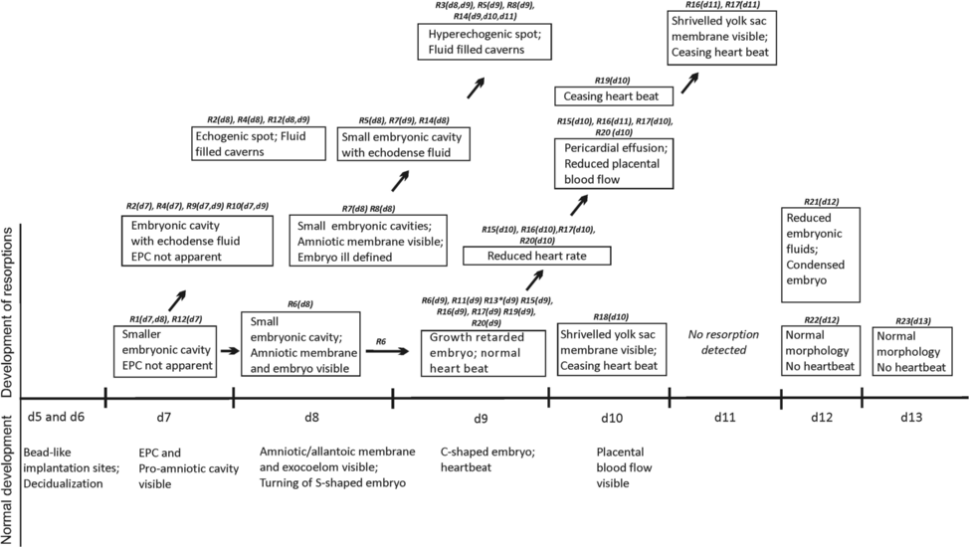
**Timeline of embryo resorption.** Ultrasonographic markers of normal development are outlined on the x-axis. The boxes on the y-axis describe the different stages of resorption. The day of detection of the different resorption stages are given in brackets for each resorption site of this study (R1-R23). The day of collection of the resorption sites is indicated by the cross symbol. Observations of follow up exams are aligned by arrows. EPC – ectoplacental cone; *embryo under resorption located outside the embryonic cavity.

The central observations are life stream scans of embryos under resorption compared with their adjacent normal litter mates. Representative scans are documented in the supplementary movies, which are much more informative than the single frames in the figure plates. Figure 1 shows an overview of the results. The major events of normal development are summarized on the abscissa and on the ordinate the major observations in the respective embryos under resorption are shown.

### Normal embryo development on days 5 to 8

**Figure 2:**
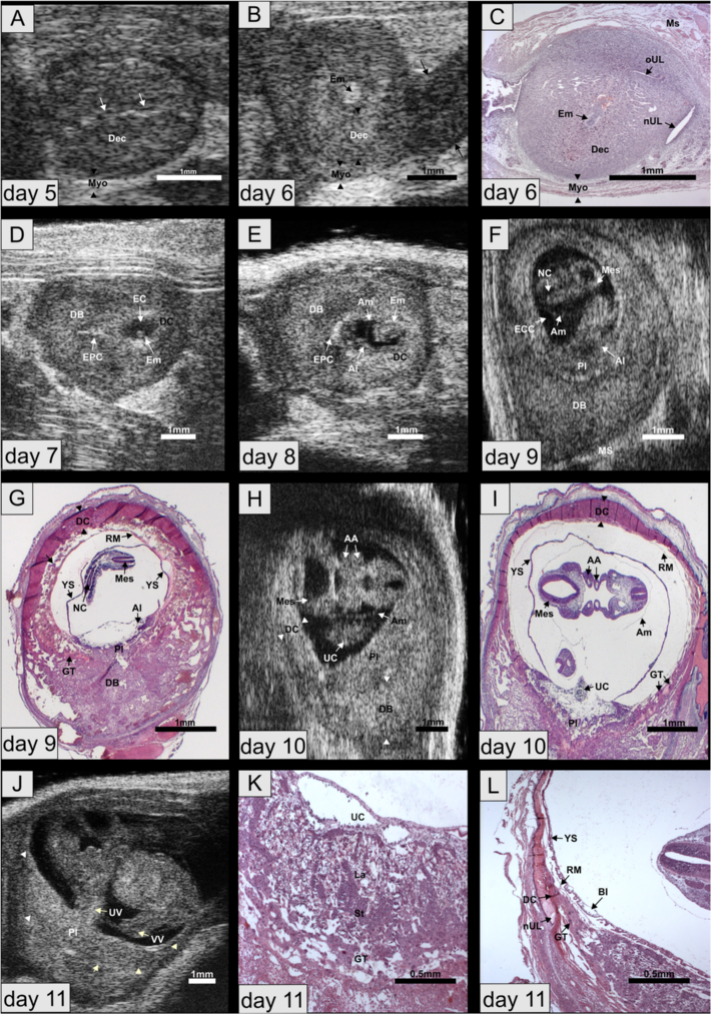
**Normal development. (A)** day 5. Note the uterine lumen between the two decidualized endometrial layers (arrows). **(B)** day 6. The myometrium has a lower echogenicity (arrowheads) compared to the decidualized endometrium. The high echodensity spot indicates the embryo. **(C)** Histological section of implantation site shown in **(B). (D)** Day 7. The embryonic cavity and the embryo proper are visible. Decidua capsularis and basalis are differentiated. **(E)** Day 8. Embryo with amnion and allantois. **(F)** Day 9. The hyperechogenic decidua capsularis is stretched out at the antimesometrial side and merges into the decidua basalis at the mesometrial side. The embryonic brain ventricles and the neural canal are visible. **(G)** Histological section of implantation site shown in **(F)**. Reichert’s membrane and yolk sac membrane are only visible in the histologic section. In vivo, these membranes are stretched out against the decidua and the placenta. **(H)** Day 10. The aortic arches and the mesencephalon are depicted. **(I)** Histology of the same embryo as in **(H). (J)** Day 11. The placenta displays hyperechogenic calcification deposits (arrowheads) at the fetomaternal boundary. **(K)** Histological section of placenta of same embryo as shown in **(J)**. The giant trophoblast is disappearing. **(L)** Histology of same embryo as in **(J** and **K)** outlining the transition zone between decidua capsularis, decidua basalis and new uterine lumen. AA - Aortic arches; Al – Allantois; Am - Amnion; DB - Decidua basalis; DC - Decidua capsularis; Dec – Decidua; EC – Embryonic cavity; ECC – Excocoelomic cavity; Em- Embryo; EPC – Ectoplacental cone; FE – Fetal erythrocytes; He – Heart; La – Labyrinth; Mes – Mesencephalon; Ms – Mesometrium; Myo – Myometrium; NC – Neural canal; nUL – new uterine lumen; oUL – old uterine lumen; RM – Reichert's membrane; Pc – Pericardium; Pl – Placenta; St – Syncytiotrophoblast; UC – Umbilical connection; UL – Uterine lumen; UV – Umbilical vessel; VV – Vitelline vessel; YS – Yolk sac.

By UBM, pregnancy could be diagnosed earliest on day 5. The implantation sites appeared as beadlike protrusions of the uterus measuring in average 1.95 mm in diameter (SD = ±0.25, N = 71), attributed to an extensive decidualization of the endometrium (Figure 2A). An additional movie file shows the normal development on days 6 to 8 (see Additional file 1: Movie S1). On **day 6**, the diameter of the implantation site had increased to 2.28 mm (SD = ± 0.39; N = 71) (Additional file 1: Movie S1, Figure 2B). The thick decidualized endometrium appeared hyperechogenic compared to the surrounding thin myometrium. Between implantation sites, endometrium and myometrium were difficult to differentiate. The uterine lumen was still visible. At that stage, the embryo was located in its yolk sac cavity (Figure 2C). On **day 7**, the embryo-maternal interface was characterized by a bright echogenic ring and the wedge-shaped ectoplacental cone protruded in the decidua basalis (Additional file 1: Movie S1, Figure 2D). The embryonic cavity measured 0.54 mm in average (SD = ± 0.20; N = 80) and could be subdivided into proamniotic, ectoplacental and exocoelom cavity. The embryo proper could not yet be reliably identified (Figure 2D). On **day 8**, the S-shaped embryo started to turn around its own axis. The allantois, which later gives rise to the umbilical vessels, was visible. It transversed the exocoelomic cavity and connected with the chorion to provide the embryonic vascular component for the chorioallantoic placenta (Additional file 1: Movie S1, Figure 2E).

### Embryo resorptions on day 7 and 8

**Figure 3:**
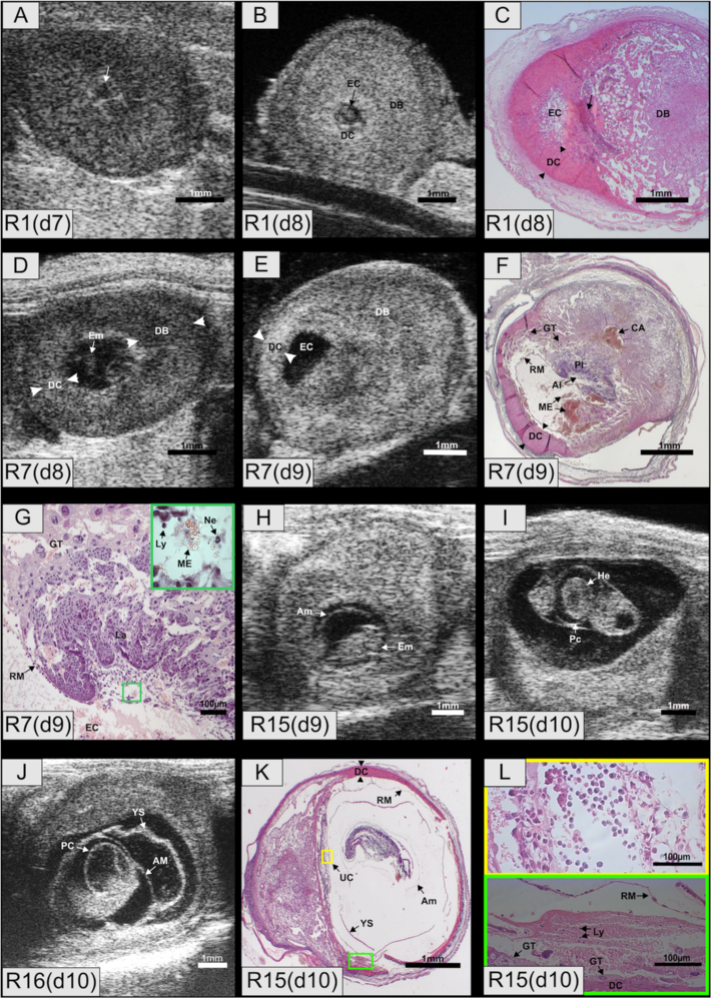
**Embryo resorptions R1, R4, R7, R15 and R16. (A)** R1, day 7. The resorption site with small embryonic cavity (arrow) lacking a well defined ectoplacental cone. **(B)** R1, day 8. Embryonic fluid with increased echodensity. **(C)** R1, day 8. Maternal hemorrhage in the giant trophoblast ring and spongious trophoblast in the transition zone. The former placental site is composed of fibrinous tissue infiltrated with maternal granulocytes (Arrow). **(D)** R7, day 8. Embryo with ill defined morphology. **(E)** R7, day 9, scanned post mortem in the water bath. The embryo has disappeared. **(F)** R7, day 9. The embryo and its membranes except for the Reichert's membrane have disappeared. The former embryonic cavity is filled with denaturated proteins. There is a massive maternal hemorrhage between the giant trophoblast and the Reichert's membrane. The central artery is filled with blood. **(G)** Placenta of R7. Embryonic erythrocytes are absent in the allantois but maternal lyomphocytes, neutrophils and erythrocytes are present. **(H)** Growth retarded embryo R15, day 9. **(I)** R15, day 10. The embryo exhibited a reduced heart rate. Pericardial effusion is evident. **(J)** R16, day 11. The heartbeat has ceased. Pericardium, amnion and yolk sac can be differentiated. **(K)** R15, day 10. All embryonic membranes are visible, but the yolk sac is devoid of blood islands. The placental morphology is normal. **(L)** R15, day 10. Magnification of areas outlined in **(K)**. Umbilical connection filled with fetal erythrocytes (yellow rectangle). Maternal blood with high proportion of immune cells between Reichert’s membrane and giant trophoblast (green rectangle). Al – Allantois; AC – Amniotic cavity; CA – Central artery; DB - Decidua basalis; DC - Decidua capsularis; EC- Embryonic cavity; Em- Embryo; GT – Giant trophoblasts; La – Labyrinth; Ly – Lymphocytes; ME - Maternal erythrocytes; Ne – Neutrophils; Pl – Placenta; RM – Reichert's membrane; UC – Umbilical connection; YS – Yolk sac.

Identification of embryo resorption was first possible on day 7 when the ectoplacental cone was visible. On day 7, seven implantation sites were suspicious for resorption (R1, R3, R2, R4, R9, R10, R12) because their embryonic cavities were smaller than in their litter mates (EC = 0.30 mm; SD = ± 0.07; N = 7) and the ectoplacental cone was not well defined. In R1 and R12 the fluid in the embryonic cavity, which could not be further differentiated, was filled with clear fluid (Figure 3A) while on day 8, the fluid was echodense (Figure 3B). In the histological section, the embryonic cavity was filled with proteinaceous material and the placentation site consisted of condensed trophoblast tissue and maternal haemorrhage (Figure 3C). In four resorptions (R2, R4, R9, R10) the embryonic fluid was already echodense on day 7. By day 8, the embryonic cavities had disappeared and the implantation sites were transformed into echodense tissue surrounded by fluid filled caverns. A hyerechogenic spot was detected in the periphery of the resorption sites. Histological analysis showed that the caverns corresponded to maternal haemorrhage in the decidua basalis and the hyperechogenic spot to fibrinoid tissue.

**On day 8**, four resorptions (R5, R6, R7 and R14) were detected on the basis of their small cavities (EC = 0.70 mm; SD = ± 0.34; N = 4) compared to 1.35 mm (SD = ± 0.35; N = 86) in the normal developing conceptuses.

In one resorption (R6) the embryo proper was visible but the embryonic cavities were smaller compared to the normal siblings. This embryo was clearly growth retarded one day later (CRL = 0.91 mm) compared to a mean CRL of 1.72 mm (SD = ± 0.13; N = 6) in its litter mates. In another resorption (R7) the embryonic cavity was also smaller and the shape of the embryo was ill defined (Figure 3D). In that resorption (R7), only the embryonic cavity was left in the follow up exam one day later (Figure 3E). Histological analysis confirmed that the embryo proper and its inner membranes had completely disappeared. Only the Reichert's membrane was found in the original embryonic cavity (Figure 3F). Between the Reichert’s membrane and the giant trophoblast layer in the transition zone of the decidua capsularis, a massive influx of maternal blood was apparent. The central artery was also filled with blood. Maternal erythrocytes, neutrophils and lymphocytes could be seen in the allantois, but no embryonic erythrocytes were detected (Figure 3G).

In the resorptions R5, R7 and R14 no embryo or embryonic membranes were visible by ultrasound. The fluid in the embryonic cavity was echodense. In the histologic section (R10) only the Reichert's membrane was left (not shown), as observed in R7. This resorption stage was transformed into the typical echodense tissue with surrounding caverns within 24 h (R5, R8 and R14).

### Normal development on days 9 to 13

**On day 9**, the originally concave embryo had assumed a convex curvature and was enclosed in its amnion. Attributed to the inversion of germ layers, the exocoelomic cavity is the main extraembryonic cavity and the yolk sac cavity consists merely of a slim slit between exocoelom and Reichert’s membrane. The yolk sac membrane was therefore not visible by UBM. Details of the embryonic morphology such as the mesencephalon and the neural tube became evident (Figure 2F) and the embryonic heartbeat could be detected (Additional file 2: Movie S2). In corresponding histological images the exocoelomic cavity had collapsed and the space between the folded visceral yolk sac membrane and the Reichert’s membrane was artificially enlarged (Figure 2G). In the yolk sac membrane, numerous blood islands had developed. Nucleated erythrocytes were evident in the allantois. The originally antimesometrial decidua, the decidua capsularis, consisted of densely packed cells and thinned towards the mesmometrial pole, where it blended into the richly vascularised mesmometrial decidua, the decidua basalis. Between the Reichert’s membrane and the decidua capsularis at the abembryonic pole, a layer of giant trophoblast formed a ring and marked the border between the placenta and the decidua basalis at the mesometrial side.

Between days **10 and 13**, the embryo considerably enlarged in size. The pericard and the heart with its atria and ventricles could be clearly distinguished by ultrasound, as well as the aortic arches (Figure 2H and I). The placenta exhibited a similar echogenicity as the decidua basalis but could be differentiated by its pulsating blood vessels and by a layer of higher echogenicity between embryonic trophoblast and maternal decidua (Figure 2J, Additional file 3: Movie S3). In the histological sections, the placenta had differentiated in its placental labyrinth, spongiotrophoblast and giant cell layer (Figure 2K). A transition zone between decidua capsularis, decidua basalis and new uterine lumen developed (Figure 2L). At this stage, the decidua capsularis and giant trophoblast cells were in the process of disappearing.

### Embryo resorptions on days 9 to 13

**On day 9**, seven resorptions (R11, R13, R15, R16, R17, R19, R20) were first identified on the basis of growth retardation. R6, that had already exhibited a smaller embryonic cavity on day 8 also showed growth retardation on day 9. The crown rump length of the embryos (mean CRL day 9 growth retarded embryos = 1.39 mm; N = 8) was comparable to a developmental stage that was reached one day earlier in the normal siblings (mean CRL day 8 normal = 0.99 mm; SD = ± 0.30; N = 77) (Additional file 2: Movie S2, Figure 3H). Placental size was also smaller in the embryos under resorption (Table 2).

**Table 2:** Crown rump-length (CRL) and placental size of normal embryos and embryo resorptions

	**day 9**	**day 10**	**day 11**	**day 12**	**day 13**
Placental width (normal embryo)	1.66 mm; SD = ± 0.32; N = 57	3.14 mm; SD = ± 0.50; N = 38	4.71 mm; SD = ± 0.63; N = 21	5.83 mm; SD = ± 0.14; N = 13	8.03 mm; SD = ± 1.36; N = 2
Placental height (normal embryo)	0.54 mm; SD = ± 0.09; N = 57	0.83 mm; SD = ± 0.15; N = 38	1.17 mm; SD = ± 0.20; N = 21	1.37 mm; SD = ± 0.14; N = 13	2.11 mm; SD = ± 0.07; N = 2
Placental width (resorption)	1.22 mm; SD = ± 0.38; N = 12	2.40 mm; SD = ± 0.58; N = 8	3.78 mm; SD = ± 0.77; N = 4	4.81 mm; SD = ± 0.98; N = 3	4.22 mm; N = 1
Placental height (resorption)	0.42 mm; SD = ± 0.09; N = 12	0.60 mm; SD = ± 0.10; N = 8	1.04 mm; SD = ± 0.21; N = 4	0.84 mm; SD = ± 0.21; N = 3	1.18 mm; N = 1
CRL (normal embryo)	2.11 mm; SD = ± 0.46; N = 72	4.01 mm; SD = ± 0.54; N = 50	5.71 mm; SD = ± 0.85; N = 29	7.38 mm; SD = ± 0.81; N = 18	9.31 mm; SD = ± 0.70; N = 5
CRL (resorption)	1.39 mm; SD = ± 0.43; N = 8	2.41 mm; SD = ± 0.75; N = 6	1.47 mm; SD = ± 0.44; N = 2	6.54 mm; SD = ± 1.48; N = 2	5.60 mm; N = 1

Within 24 hours, the growth retarded embryos exhibited bradycardia (92 bpm in resorption prone embryos versus 130 bpm in normal siblings) and pericardial effusion (Additional file 3: Movie S3, Figure 3I). The size of the heart corresponded to that of a normally developing embryo and was therefore proportionally bigger to embryonic body size. Interestingly the resorption prone embryos were still able to develop further, albeit at a reduced rate (Additional files 3, 4 and 5, Figure 3H and I). Due to the loss of embryonic fluids, the formerly expanded exocoelomic cavity deflated and the folded yolk sac membrane became visible by UBM (Additional file 4: Movie S4, Figure 3J). One resorption prone embryo (R15) that was first detected on day 9 (Figure 3H) showed a reduced heart beat on day 10 (Figure 3I). It was collected on day 10 when it was still alive but its heartbeat was barely detectable. In the corresponding histological sections all membranes were identified (Figure 3K) though the yolk sac membrane was lacking the typical blood islands. In the transition zone, maternal blood had accumulated between the Reichert’s membrane and the decidua capsularis, which was still delineated by giant trophoblast cells (Figure 3L). The umbilical vessel was filled with embryonic erythrocytes (Figure 3L), which underlined the previous ultrasonographic observation of a faint heartbeat (Additional file 5: Movie S5). Surprisingly, the cells of the embryo proper showed signs of necrosis to a great extent.

**Figure 4:**
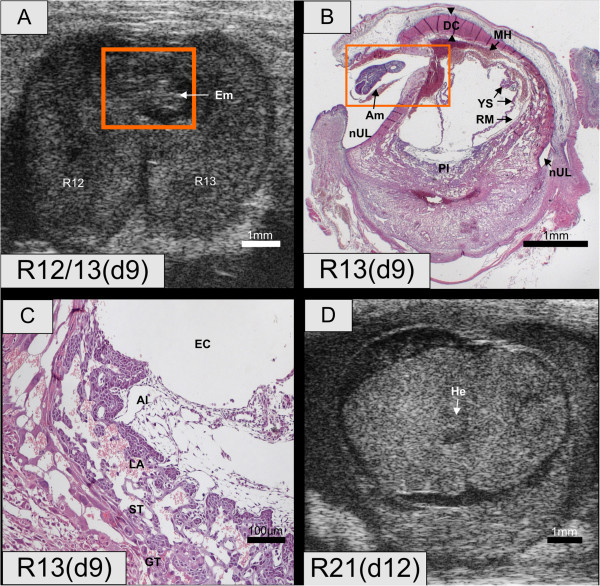
**Embryo resorptions R12, R13 and R21. (A)** R12 and R13, day 9. In R12, the embryo is no longer visible. In R13, the embryo is still present but it is located in the uterine lumen. **(B)** R13, day 9. The decidua capsularis is ruptured. The embryo is encased in its amnion but outside the yolk sac membrane and Reichert’s membrane, which are still located in the original embryonic cavity. The yolk sac membrane exhibits blood islands and is folded due to fluid loss. Between Reichert's membrane and yolk sac membrane, there is maternal hemorrhage. **(C)** The placental barrier is intact. Blood spaces filled with maternal blood are evident as well as embryonic blood vessels containing nucleated erythrocyte. **(D)** R21, day 12. There was no visible heartbeat and less fluid in the embryonic cavity. Al – Allantois; Am – Amnion; DC – Decidua capsularis; EC – Embryonic cavity; Em – Embryo; GT – Giant trophoblasts; MH - Maternal hemorrhage; nUL – New uterine lumen; PE – Pericardial effusion; Pl – Placenta; RM – Reichert's membrane; UC – Umbilical connection; YS – Yolk sac.

In the next phase of the resorption process, the heartbeat finally ceased. Histological analysis showed that the resorption process continued with the necrosis of the embryo proper, which was still surrounded by its membranes. In two cases (R13, R16), the embryo was located in the uterine lumen due to a rupture of the decidua capsularis (Figure 4A and B, Additional file 4: Movie S4). In the histological section of R13, yolk sac and Reichert's membrane were found inside the embryonic cavity. The exteriorized embryo was surrounded by its amniotic membrane. The morphology of its placenta was unsuspicious (Figure 4C). In our study, there were no indications for a first detection of the resorption process on day 11.

**On day 12**, two embryos that were unsuspicious on the previous day were found dead (R21, R22). One embryo (R21) showed reduced embryonic fluids and condensed embryonic tissue (Figure 4D). On day 13 an embryo that presented a normal morphology had no visible heart beat (R23).

In conclusion, the process of embryo resorption is characterized by four distinct phases: in the first phase, growth retardation is observed which manifests in the reduced size of the embryonic cavities and the delayed developmental stage of the embryo itself. In the second phase, the embryo exhibits a reduced, sometimes irregular heartbeat, reduced placental blood flow, detachment of the yolk sac membrane from the outer Reichert’s membrane and pericardial effusion which subsequently results in stalling of the heartbeat. In the third phase, first the embryo disintegrates, then its inner membranes disappear and finally the placental integrity is lost. In the final stage of resorption, the implantation site is characterized by hypoechogenic caverns and a central, hyperechogenic spot which correspond to maternal haemorrhage and fibrinoid tissue, respectively. The time course of the outlined resorption stages and the respective morphological characteristics vary according to gestational age as outlined above.

## Discussion

In our study we described the *in vivo* process of murine embryo resorption using UBM and correlated our ultrasound data to histology. The normal development served as a reference for the successful *in vivo* detection of embryo resorption.

The resorption followed a specific pattern, independent from the time in gestation when the resorption process started. The first sign to presage resorption prone embryos was delayed development or growth retardation. On the basis of our study growth retardation can be strongly associated with the resorption process, even though a considerable variability in developmental stage within one litter has been demonstrated in the post mortem study of Thiel et al. [25]. The developmental difference observed in the study of Thiel et al. varied by almost one day. However, since these observations rely on post mortem findings, the subsequent development of the smaller embryos was not documented. We assume that some of embryos which exhibited the least development were in fact prone for resorption. Measurements of CRL on day 9 from our study support this possibility since the gestational age difference between embryos of the same litter was not greater than half a day if we excluded the resorption prone embryos.

In early pregnancy stages, growth retardation manifested in smaller embryonic cavities and an undifferentiated shape of the embryo itself. In our study, a smaller embryonic cavity suggestive for resorption could be first observed on day 7.

On day 8, resorption prone conceptuses could be identified by their smaller size. In some cases, the embryo itself first developed but its morphology was ill defined and it then disappeared within 24 hours. The detection of embryos under resorption that early in gestation is therefore highly dependent on the level of experience of the sonographer, considering that the morphological changes are delicate and occur at a fast rate. It seems that with advancing development, the time period between the first signs of growth retardation and death of the embryo is elongated, making it more likely to identify living but resorption prone embryos. Beginning on day 9, when the heart beat was reliably detectable, the second stage of the resorption process could be visualized. Here, the dysfunction of the embryonic circulation manifested in bradycardia, reduced placental perfusion, and pericardial edema.

Another finding, the visualization of the shrivelled yolk sac membrane by UBM which is not evident in normal embryos might account for the fact that the limited and finally ceased production of embryonic fluids reduces the physiological turgor of the exocoelomic cavity. This eventually leads to an artificial increase of the yolk sac cavity.

The third phase of the resorption process implies the death of the embryo. In other studies, the death of the embryo is defined by a ceasing heartbeat. The heart of the mouse starts to beat between day 8 and 9 [26,27]. Diagnosis of embryonic death on the basis of heart action is therefore not possible prior this day. Theoretically, the determination of the exact time point of embryonic death based on the absence of a heartbeat would require permanent live ultrasound scanning. In our study, bradycardia, arrhythmia and pericardial edema in growth retarded conceptuses preceded the final cessation of heart function. After observation of these markers, ultrasound examinations can be performed twice daily to delineate the time window of death. However, one has to consider that ceasing of the heartbeat might in fact not be the appropriate marker for embryonic death. We showed that one embryo under resorption, which still exhibited a faint heartbeat, already showed severe necrosis of the cells of the embryo proper. This finding shows that the border between the end of life of an individual is equally fluent and difficult to define as its beginning. In early gestation, it is even harder to narrow down the period of death since the embryo proper only begins to develop and its heart is not yet beating. Death in that developmental stage occurs at the cellular level only and is reflected in arrested development of the conceptus, accompanied by an increased echogenicity of embryonic fluids as seen by high frequency ultrasound.

In the fourth stage the conceptus dissolves and is subject to haemorrhage and necrosis. The final resorption stage consists of fibrinoid, condensed scar tissue which persists for an extended period of time. In post mortem studies, this is the stage where the resorption site is identified macroscopically in the exteriorized uterus [11,28-30]. By ultrasound, this final stage of resorption observed in early pregnancy was characterised by a high echodensity spot. We documented similar high-echodensity spots along the ectoplacental cone and in the placenta at the embryo-maternal border in normal conceptuses. In a study of Akirav et al., these high density foci have been identified as calcium deposits [31]. The concretions most likely originate from dystrophic calcification processes in dysfunctional cells where the active calcium transport is impaired. They can therefore be considered as a marker for the last stages of apoptosis and necrosis.

This process seems to originate from the embryo itself, since we always observed first the death of the embryo, then its dissolution, and then the disappearance of its inner membranes. These observations have also been made in an ultrasound study on embryo resorption in the European brown hare [32]. In human reproductive medicine, an anembryonic gestational sac is considered as an ultrasonographic marker for embryonic demise [33]. High levels of alpha-fetoprotein of yolk sac origin in the maternal circulation are indicative for an early death of the embryo which was resorbed before it became ultrasonographically detectable [34].

In our study, the Reichert’s membrane which is unique to rodents and acts as a filter between embryonic and maternal tissue [35], was the last membrane to disappear. Together with the finding that the placenta was morphologically unsuspicious these observations support the hypothesis that death is triggered within the embryo itself. By means of cell competition, embryonic cells can compare their fitness with that of neighbouring cells resulting in apoptosis of the less fitter cells [36]. This mechanism has been demonstrated to play a crucial role in the selection of mouse embryonic epiblast [37]. If that cell competition becomes unbalanced, it could result in embryonic death. There seems to be a higher selection pressure on the long lived epiblast cells than on the short lived cells of the extraembryonic membranes [37], reflecting our finding that the membranes undergo resorption only after the embryo has already disappeared.

In knockout mice, the depletion of specific genes may result in a characteristic embryonic phenotype [20,24] which can be further examined with additional methods such as hybridization and immunohistochemistry. Longitudinal UBM examinations will enable to determine the exact stage at which certain genes need to be expressed to ensure healthy embryo development and survival.

The monitoring of embryo development by UBM will also be useful in the field of epigenetics, where certain environmental factors acting on the adult individual influence the gene expression and intra-uterine development of their offspring [38,39]. The use of UBM to detect embryo resorptions will significantly reduce the number of experimental animals in studies investigating embryo failure for two reasons: first, pregnant animals can be reliably detected from day 5 onward and non-pregnant animals can be saved. Second, embryo resorptions can be identified *in vivo* at an early stage enabling the sacrifice of the experimental animal at the appropriate time.

## Conclusions

In our study we have shown that UBM is a useful tool to detect resorption prone embryos and to follow their involution process over time. With this method, resorption prone embryos can be specifically targeted and harvested before the onset of decomposition. This is particularly important for the study of embryo-maternal immune reactions, where the specific maternal immune response towards the dying embryo must be differentiated from a general immune reaction necessary to clear the uterus from apoptotic tissue. Furthermore, the morphology of the placenta and extraembryonic membranes can be evaluated *in vivo* over a period of time before its integrity is compromised by dissection.

## Competing interests

The authors have no competing interests.

## Authors’ contributions

TBH, LEF and BD developed the study design. LEF and BD performed ultrasound imaging and retrospective analysis. LEF, AAK and BD evaluated the histological sections. LEF and BD wrote the manuscript. All authors read and approved the final manuscript.

## Supplementary Material

Additional file 1: Movie S1 Normal development day 6–8. On day 6, the implantation site is visible as a bulge of the uterus. The endometrial layers are seen as a hyperechogenic, white line. On day 7, the ectoplacental cone and the proamniotic cavity become evident. On day 8, the embryo is encased in its amnion and the allantois traverses the exocoelom. The ectoplacental cone invades the mesometrial decidua basalis. Click here for file

Additional file 2: Movie S2 R16 and R17 day 9. In the normal embryo, head and rump can be differentiated and the heartbeat is evident. The umbilical cord attaches to the placenta. The resorption prone embryos R16 and R17 display the same morphological features but are visibly smaller compared to the normal embryo. Click here for file

Additional file 3: Movie S3 R16 and R17 day 10. The normal embryo displays a pulsating heart with atria and ventricles enclosed in the pericardium. The blood flow from the umbilical cord to the placenta is visible. The embryo-maternal interface is characterized by calcifications between the trophoblast and the placenta. The resorption prone embryo R16 is visibly smaller than its normal sibling. However, its heart rate is not yet reduced. The resorption prone embryo R17 shows growth retardation, pericardial effusion and a reduced heartbeat. Click here for file

Additional file 4: Movie S4 R16 and R17 day 11, first and second scan. The normal embryo has increased in size. In contrast, the R16 is now clearly in the process of resorption: the heart is compressed by pericardial effusion and a heartbeat is barely detectable. The yolk sac is visible as a shrivelled membrane after having lost its close connection to the Reichert's membrane and underlying maternal mucosa. R17 has died. The amniotic cavity is small and the yolk sac has also lost its balloon like shape, resulting in a shriveled yolk sac membrane. In the second scan, 3 hours later, the morphology of the normal embryo is not altered. In R16, a heartbeat cannot be detected. The embryo of R17 has completely lost its original morphology and its tissue looks condensed. The embryo seems to be outside its original cavity in the uterine lumen. During collection of the resorption site, the embryo was lost. Only the Reichert's membrane was found in the uterine lumen. Click here for file

Additional file 5: Movie S5 R15 day 9 and 10. On day 9 the resorption prone embryo R15 shows visible growth retardation. Compared to its normal sibling, the morphology is poorly defined. On day 10, the resorption prone embryo has increased in size and its heart can be differentiated. The pericard is filled with excess fluid and its heart rate is greatly reduced as illustrated by color doppler flow. Click here for file

## References

[B1] Macklon NS Geraedts JP Fauser BC Conception to ongoing pregnancy: the ‘black box’ of early pregnancy loss Hum Reprod Update 2002 8 4 333 343 10.1093/humupd/8.4.333 12206468

[B2] Bulletti C Flamigni C Giacomucci E Reproductive failure due to spontaneous abortion and recurrent miscarriage Hum Reprod Update 1996 2 2 118 136 10.1093/humupd/2.2.118 9079408

[B3] Diskin MG Parr MH Morris DG Embryo death in cattle: an update Reprod Fertil Dev 2011 24 1 244 251 22394965 10.1071/RD11914

[B4] Andrabi SM Maxwell WM A review on reproductive biotechnologies for conservation of endangered mammalian species Anim Reprod Sci 2007 99 3–4 223 243 16919407 10.1016/j.anireprosci.2006.07.002

[B5] Simpson J Bombard A Chromosomal abnormalities in spontaneous abortion: frequency, pahtology and genetic counseling Spontaneous Abortion 1991 London: Blackwell 51

[B6] Reynolds LP Caton JS Redmer DA Grazul-Bilska AT Vonnahme KA Borowicz PP Luther JS Wallace JM Wu G Spencer TE Evidence for altered placental blood flow and vascularity in compromised pregnancies J Physiol 2006 572 1 51 58 16469783 10.1113/jphysiol.2005.104430 PMC1779650

[B7] Zenclussen AC Gerlof K Zenclussen ML Sollwedel A Bertoja AZ Ritter T Kotsch K Leber J Volk HD Abnormal T-cell reactivity against paternal antigens in spontaneous abortion - Adoptive transfer of pregnancy-induced CD4 (+) CD25 (+) T regulatory cells prevents fetal rejection in a murine abortion model Am J Pathol 2005 166 3 811 822 10.1016/S0002-9440(10)62302-4 15743793 PMC1602357

[B8] Farese RV Jr Ruland SL Flynn LM Stokowski RP Young SG Knockout of the mouse apolipoprotein B gene results in embryonic lethality in homozygotes and protection against diet-induced hypercholesterolemia in heterozygotes Proc Natl Acad Sci U S A 1995 92 5 1774 1778 10.1073/pnas.92.5.1774 7878058 PMC42602

[B9] Chang H Huylebroeck D Verschueren K Guo Q Matzuk MM Zwijsen A Smad5 knockout mice die at mid-gestation due to multiple embryonic and extraembryonic defects Development 1999 126 8 1631 1642 10079226 10.1242/dev.126.8.1631

[B10] Passey RJ Williams E Lichanska AM Wells C Hu S Geczy CL Little MH Hume DA A null mutation in the inflammation-associated S100 protein S100A8 causes early resorption of the mouse embryo J Immunol 1999 163 4 2209 2216 10438963

[B11] Murphy SP Fast LD Hanna NN Sharma S Uterine NK cells mediate inflammation-induced fetal demise in IL-10-null mice J Immunol 2005 175 6 4084 4090 10.4049/jimmunol.175.6.4084 16148158

[B12] Lin Y Nakashima A Shima T Zhou X Saito S Toll-like receptor signaling in uterine natural killer cells–role in embryonic loss J Reprod Immunol 2009 83 1–2 95 100 19889464 10.1016/j.jri.2009.09.004

[B13] Clark DA Petitbarat M Chaouat G How should data on murine spontaneous abortion rates be expressed and analyzed? Am J Reprod Immunol 2008 60 3 192 196 10.1111/j.1600-0897.2008.00612.x 18652577

[B14] Roellig K Drews B Goeritz F Hildebrandt TB The long gestation of the small naked mole-rat (Heterocephalus glaber Ruppell, 1842) studied with ultrasound biomicroscopy and 3D-ultrasonography PLoS One 2011 6 3 e17744 10.1371/journal.pone.0017744 21408185 PMC3049790

[B15] Schroeder K Drews B Roellig K Menzies BR Goeritz F Hildebrandt TB In vivo tissue sampling of embryonic resorption sites using ultrasound guided biopsy Theriogenology 2011 76 4 778 784 10.1016/j.theriogenology.2011.03.012 21601265

[B16] Drews B Ringleb J Waurich R Hildebrandt TB Schroder K Roellig K Free blastocyst and implantation stages in the European brown hare: correlation between ultrasound and histological data Reprod Fertil Develop 2012 25 6 866 878 10.1071/RD12062 22953725

[B17] Mu J Slevin JC Qu D McCormick S Adamson SL In vivo quantification of embryonic and placental growth during gestation in mice using micro-ultrasound Reprod Biol Endocrinol 2008 6 34 10.1186/1477-7827-6-34 18700008 PMC2527569

[B18] Zhou YQ Foster FS Qu DW Zhang M Harasiewicz KA Adamson SL Applications for multifrequency ultrasound biomicroscopy in mice from implantation to adulthood Physiol Genomics 2002 10 2 113 126 12181368 10.1152/physiolgenomics.00119.2001

[B19] Pallares P Gonzalez-Bulnes A Use of ultrasound imaging for early diagnosis of pregnancy and determination of litter size in the mouse Lab Anim 2009 43 1 91 95 10.1258/la.2008.007139 19001063

[B20] Laissue P Burgio G L'Hote D Renault G Marchiol-Fournigault C Fradelizi D Fellous M Serres C Montagutelli X Monget P Vaiman D Identification of Quantitative Trait Loci responsible for embryonic lethality in mice assessed by ultrasonography Int J Dev Biol 2009 53 4 623 629 10.1387/ijdb.082613pl 19488966

[B21] Phoon CK Turnbull DH Ultrasound biomicroscopy-Doppler in mouse cardiovascular development Physiol Genomics 2003 14 1 3 15 12824473 10.1152/physiolgenomics.00008.2003

[B22] Phoon CK Ji RP Aristizabal O Worrad DM Zhou B Baldwin HS Turnbull DH Embryonic heart failure in NFATc1−/− mice: novel mechanistic insights from in utero ultrasound biomicroscopy Circ Res 2004 95 1 92 99 10.1161/01.RES.0000133681.99617.28 15166096

[B23] Phoon CK Aristizabal O Turnbull DH 40 MHz Doppler characterization of umbilical and dorsal aortic blood flow in the early mouse embryo Ultrasound Med Biol 2000 26 8 1275 1283 10.1016/S0301-5629(00)00278-7 11120365

[B24] Vatin M Burgio G Renault G Laissue P Firlej V Mondon F Montagutelli X Vaiman D Serres C Ziyyat A Refined mapping of a quantitative trait locus on chromosome 1 responsible for mouse embryonic death PLoS One 2012 7 8 e43356 10.1371/journal.pone.0043356 22916247 PMC3420870

[B25] Thiel R Chahoud I Jurgens M Neubert D Time-dependent differences in the development of somites of four different mouse strains Teratog Carcinog Mutagen 1993 13 6 247 257 10.1002/tcm.1770130602 7903826

[B26] Srinivasan S Baldwin HS Aristizabal O Kwee L Labow M Artman M Turnbull DH Noninvasive, in utero imaging of mouse embryonic heart development with 40-MHz echocardiography Circulation 1998 98 9 912 918 10.1161/01.CIR.98.9.912 9738647

[B27] Ji RP Phoon CK Aristizabal O McGrath KE Palis J Turnbull DH Onset of cardiac function during early mouse embryogenesis coincides with entry of primitive erythroblasts into the embryo proper Circ Res 2003 92 2 133 135 10.1161/01.RES.0000056532.18710.C0 12574139

[B28] Kusakabe K Naka M Ito Y Eid N Otsuki Y Regulation of natural-killer cell cytotoxicity and enhancement of complement factors in the spontaneously aborted mouse placenta Fertil Steril 2008 90 4 Suppl 1451 1459 18068164 10.1016/j.fertnstert.2007.07.1331

[B29] Duclos AJ Haddad EK Baines MG Presence of activated macrophages in a murine model of early embryo loss Am J Reprod Immunol 1995 33 5 354 366 10.1111/j.1600-0897.1995.tb00904.x 7576117

[B30] Clark DA Foerster K Fung L He W Lee L Mendicino M Markert UR Gorczynski RM Marsden PA Levy GA The fgl2 prothrombinase/fibroleukin gene is required for lipopolysaccharide-triggered abortions and for normal mouse reproduction Mol Hum Reprod 2004 10 2 99 108 10.1093/molehr/gah013 14742694

[B31] Akirav C Lu Y Mu J Qu DW Zhou YQ Slevin J Holmyard D Foster FS Adamson SL Ultrasonic detection and developmental changes in calcification of the placenta during normal pregnancy in mice Placenta 2005 26 2–3 129 137 15708114 10.1016/j.placenta.2004.05.010

[B32] Schroeder K Drews B Roellig K Goeritz F Hildebrandt TB Embryonic resorption in context to intragestational corpus luteum regression: A longitudinal ultrasonographic study in the European brown hare (Lepus europaeus PALLAS, 1778) Theriogenology 2013 76 4 778 784 23773690 10.1016/j.theriogenology.2013.05.010

[B33] Odeh M Tendler R Kais M Grinin V Ophir E Bornstein J Gestational sac volume in missed abortion and anembryonic pregnancy compared to normal pregnancy J Clin Ultrasound 2010 38 7 367 371 20533447 10.1002/jcu.20713

[B34] Jauniaux E Gulbis B Jurkovic D Gavriil P Campbell S The origin of alpha-fetoprotein in first-trimester anembryonic pregnancies Am J Obstet Gynecol 1995 173 6 1749 1753 10.1016/0002-9378(95)90421-2 8610756

[B35] Salamat M Gotz W Horster A Janotte B Herken R Ultrastructural localization of carbohydrates in Reichert's membrane of the mouse Cell Tissue Res 1993 272 2 375 381 10.1007/BF00302742 8513488

[B36] de la Cova C Abril M Bellosta P Gallant P Johnston LA Drosophila myc regulates organ size by inducing cell competition Cell 2004 117 1 107 116 10.1016/S0092-8674(04)00214-4 15066286

[B37] Claveria C Giovinazzo G Sierra R Torres M Myc-driven endogenous cell competition in the early mammalian embryo Nature 2013 500 7460 39 44 10.1038/nature12389 23842495

[B38] Simmons RA Developmental origins of adult disease Pediatr Clin North Am 2009 56 3 449 466 10.1016/j.pcl.2009.03.004 19501686 PMC3357632

[B39] Jammes H Junien C Chavatte-Palmer P Epigenetic control of development and expression of quantitative traits Reprod Fertil Develop 2012 23 1 64 74 10.1071/RD10259 21366982

